# Medical Cost Trajectories and Onsets of Cancer and NonCancer Diseases in US Elderly Population

**DOI:** 10.1155/2011/857892

**Published:** 2011-06-01

**Authors:** Igor Akushevich, Julia Kravchenko, Lucy Akushevich, Svetlana Ukraintseva, Konstantin Arbeev, Anatoliy I. Yashin

**Affiliations:** ^1^Center for Population Health and Aging, Duke University, Durham, NC 27708, USA; ^2^Duke Cancer Institute, Duke University, Durham, NC 27705, USA

## Abstract

Time trajectories of medical costs-associated with onset of twelve aging-related cancer and chronic noncancer diseases were analyzed using the National Long-Term Care Survey data linked to Medicare Service Use files. A special procedure for selecting individuals with onset of each disease was developed and used for identification of the date at disease onset. Medical cost trajectories were found to be represented by a parametric model with four easily interpretable parameters reflecting: (i) prediagnosis cost (associated with initial comorbidity), (ii) cost of the disease onset, (iii) population recovery representing reduction of the medical expenses associated with a disease since diagnosis was made, and (iv) acquired comorbidity representing the difference between post- and pre diagnosis medical cost levels. These parameters were evaluated for the entire US population as well as for the subpopulation conditional on age, disability and comorbidity states, and survival (2.5 years after the date of onset). The developed approach results in a family of new forecasting models with covariates.

## 1. Introduction

Determining the national trends in health, disease burden, and associated health expenditures in the US population with growing proportions of elderly individuals is a major public health concern and an important issue for policymakers and governmental institutions. Aging-related deterioration in health involves an important economical component—that is, medical costs associated with disease treatment and rehabilitation strategies to minimize the effect of disability on economics. To forecast it, it is important to understand the key factors driving progression of aging-related cancer and noncancer chronic diseases and the associated medical costs of health care providers such as Medicare and Medicaid. In 2009, 46.3 million people were covered by Medicare: 38.7 million of them were 65 years and older, and 7.6 million were disabled [1]. By 2031, the enrollment in Medicare is expected to reach 77 million, when the baby-boomers generation is fully enrolled [2]. The Medicare program covers 95% of the nation's aged population [3], therefore, the prediction of future Medicare costs is an important component of health care planning. Medicare costs result from summarizing medical costs for each individual enrolled in the system; individual costs deal with expenditures associated with the disease onset and the consequences of aging-related chronic conditions.

Detailed and comprehensive analysis has recently been performed to investigate the aggregate spending on the Medicare Part A and B programs for the U.S. elderly population in their final years of  life. The relationships between Medicare costs and disability and morbidity were considered by Goldman [4]: the Future Elderly Model (FEM) was developed to predict the medical costs and health status for the elderly. However, the “portrait” of individual histories of changing health status, and the relationships of such changes with dynamics of Medicare expenditures as the person ages, were not investigated in detail. The important topics requiring further analyses are the costs associated with specific aging-related cancer and noncancer chronic diseases, the influence of disease onset on individual medical cost trajectories, the behavior of individual health trajectories in presence of comorbid and concurrent disorders, how analysis of structure of medical expenditures can help healthcare providers find ways for controlling the costs, and to what extent cumulative individual medical costs can determine future changes in health status. Studies of such problems appear episodically. New results in this area will open new possibilities for population health and medical cost forecasting, allowing for empirical base developing for assessing the impact of new biotechnologies on increasing the years of minimally disabled life [5]. 

The modern models of population health status forecasting with associated medical costs include three essential components or submodels: (i) the model of medical cost projections conditional on health state, (ii) health state projections, and (iii) description of initial health state of a cohort to be projected [4, 6–8]. Two major effects should be taken into account while modeling the medical cost projections: dynamics of the medical costs during the time periods comprising the date of onset of chronic diseases and the medical cost increase in the last years of life. In this paper, we investigated and modeled the first of these two effects (the latter was extensively investigated in the literature—see [9–11]). The analyses of medical cost trajectories in the time period of health change are capable of revealing many substantive properties of Medicare expenditures for the entire U.S. elderly population, as well as for subpopulations conditional on a specific heath state (e.g., described by disability and/or comorbidity indices). Besides, it could generalize the approaches known as life tables with covariates [12, 13] resulting in a family of new forecasting models with a covariate such as comorbidity index or the medical cost.

Thus, this study is focused on developing a model capable of a quantitative description of the relationships between individual cost trajectories around the onset of an aging-related cancer and noncancer chronic diseases. The model is supposed to have demographically interpretable parameters and to serve as a building block in constructing a more precise and comprehensive forecasting model of medical costs (including Medicare spending) on population level. The underlying methodological idea was to aggregate the health state information into a single (or several) covariate(s) which can be determinative in predicting the risk of a health event (e.g., disease incidence) and whose dynamics could be determined by the model assumptions. An advantage of such an approach is in its substantial reduction of degrees of freedom compared with existing forecasting models—as a result, the forecasting models in continuous time estimated with the limited information might become a close achievement.

## 2. Data and Methods

### 2.1. National Long-Term Care Survey (NLTCS), Medicare Files of Service Use, and Medical Cost

The primary data to be analyzed are the six waves of the NLTCS [14] spanning the period from 1982 to 2004/5 linked to Medicare data. Two of the six waves, namely, cohorts of 1994 and 1999, are used in the analysis. These specific waves were chosen primarily because the high quality Medicare follow-up data are available only starting from 1991, and also because the complete 5-year follow-up after the NLTCS interview for later than 1991 is accessible only for these two waves. The NLTCS uses a sample of individuals drawn from the national Medicare enrollment files. The NLTCS provides the reported data on hundreds of variables including age, sex, and (instrumental) activities of daily living (ADL/IADL) allowing for disability measurements. The same data collection agency, the U.S. Census Bureau, was employed for collecting data over all of the waves—so, the training methods and materials, survey administration and management procedures, field operations, computer processing, and editing procedures were consistent across the surveys. In addition to these, the high response rates (95%) across all NLTCS waves allowed to minimize the bias in trend estimates. The results of interest (i.e., parameters describing medical cost trajectories) are similar for cohorts formed in 1994 and 1999 (that will be further discussed in Section 4). The 1982 to 2004 NLTCS files include information on 49,258 different individuals, and 34,077 of them were followedup in 1994–2004. The national population estimates were produced using screener weights released with the NLTCS.

All individuals in the NLTCS are continuously tracked for Medicare Parts A and B service use. Thus, for all persons we have continuous records of Medicare service use from 1991 or since the person passed age 65 after 1990 and until death. These records are available for each institutional (inpatient, outpatient, skilled nursing facility, hospice, or home health agency) and noninstitutional (Carrier-Physician-Supplier and durable medical equipment providers) claim type.

### 2.2. Date of Disease Onset Definitions

The date of disease onset was identified using information collected in the Medicare Claims files. Unlike mortality, the onset time of chronic disease is difficult to define with precision due to the variety of disease-specific criteria for onset/incident case identification (e.g., incidence case for ACHD, fatal incidence for stroke) used in clinical practice and epidemiological and population-based analyses. Thus, there is a certain arbitrariness in defining the date of onset which can be used for constructing a unified definition of date of onset appropriate for population studies. The scheme used in this paper resulted from an overview of several approaches to such definition [15–17]. The unified scheme is useful for comparative analyses of the effects of different diseases on the medical cost and is also appropriate for prediction purposes.

The following scheme was used to reconstruct the ages at onsets of all diseases from the Medicare service use data. First, the individual medical histories of the applicable disease were reconstructed from the Medicare files combining all records with their respective ICD-9 codes. The following ICD-9 codes were used: acute coronary heart disease (410.xx, 411.xx, 413.xx), stroke (431.xx, 433.x1, 434.x1, 436.xx), ulcer (531.xx, 532.xx, 533.xx, 534.xx), breast cancer (174.xx), prostate cancer (185.xx), melanoma (172.xx), lung cancer (162.xx), colon cancer (153.xx), diabetes (250.xx), asthma (493.xx), Parkinson's disease (332.xx), and Alzheimer's disease (331.0, 290.1). Then, the individuals with the history of a considered disease before the date of interview in 1994 or in 1999 were excluded from the cohort. Because detailed individual records in Medicare files are available from 1991, we have a sufficient period of time to reject the prevalence cases. The numbers of individuals in the pooled cohort without the prevalent cases for each disease are shown in Table 1. A date of a Medicare record (referred to as “*this record*” below in the (i) and (ii)) is identified with the date of onset of applicable condition if both conditions mentioned below are met:

- *this record* is the earliest record with respective ICD code as a primary diagnosis in one of four Medicare sources (inpatient care, outpatient care, physician services, and skilled nursing facilities).
- in addition to *this record*, there is another record with its respective ICD code as the primary diagnosis from one of the four Medicare sources listed in (i), which appeared with a date different from the date of *this record* and no later than 0.3 years after *this record*.

This definition of the age at disease onset fixes a definition of disease incidence. Since the date of onset of a certain chronic disease is a quantity not defined as precise as mortality, some assumptions are required to identify the date of onset from individual records collected in administrative data. The specifications used in this paper (e.g., choice of the four Medicare sources in item (i) and time period of 0.3 year in item (ii)) are in accordance with the general practice of reconstruction of the date at onset from Medicare data [15, 17].

### 2.3. Medical Cost Trajectories

For each disease, the individuals whose date of onset occurred during the 5 years after the date of interview were selected (see Table 1). Then, they were stratified into subgroups by certain indices. The following variables were used for stratification: Charlson comorbidity index (calculated using Medicare data), disability index (measured in screener interview, see [18]), survival status in 2.5 years, and age at diagnosis. The Charlson comorbidity index was calculated according to the specifications described in Charlson et al. [19] and Quan et al. [20], as a weighted sum of chronic conditions appeared in individual medical records during the year prior to the date of interview. The seventeen groups of chronic conditions contribute to the Charlson index, and their weights are related to their relative risks of death. Disability index is constructed from the screener questionnaire of elicited information on six activities of daily living (ADL, e.g., difficulty eating) and eight instrumental activities of daily living questions (IADL, e.g., difficulties with light housework; laundry) [21]. The used index is a variable with three categories: (i) nondisabled, (ii) IADL only or/and 1-2 ADLs, and (iii) 3–6 ADLs. 

For each of the twelve disease-specific groups and strata-defined subgroups, means and standard errors of the distributions of medical cost spending per month per capita were estimated within 20 months before and after disease onset. The empirical estimates demonstrated that 20 months could be a sufficient period of time for “stabilization” after disease onset by reaching a plateau in the mean of the medical cost trajectories. In our study, these month patterns (or medical cost trajectories) were subject to analysis, mutual comparison, and modeling. The right censoring effects were taken into account for the cohort of patients not surviving 2.5 years after diagnoses. All costs were presented in terms of the dollar value from year 2000, being adjusted for inflation using the Medical Care Consumer Price Index provided by the Bureau of Labor Statistics [22].

## 3. Results

**Figure 1:**
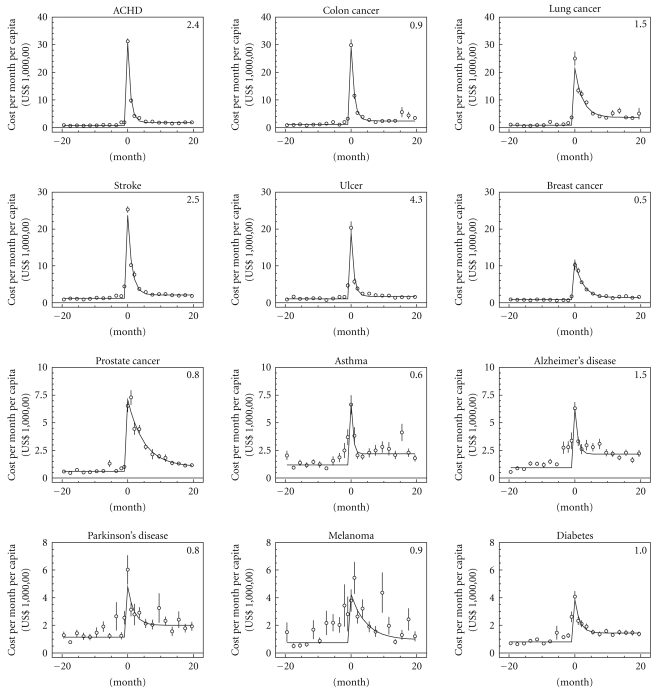
Empirical estimates (dots) and model predictions (solid lines) of cost per month per capita. The diseases are ordered according to the cost of onset. Values in the right upper corners of all plots are *X* ^2^ per degree of freedom calculated as *df* ^−1^∑_*i*=20_^20^(*C*_*i*_ − *C*(m))^2^/*σ*_*i*_^2^, where *C*_*i*_ and *σ*_*i*_ are estimated mean and standard error of medical cost per month (for presentation purposes they were aggregated into two-month groups); *df* denoted the degree of freedom calculated as the difference between the number of measured points (41) and the number of estimated parameters (4). Note that the scale of the vertical axes is not the same for different rows of plots.

**Figure 2:**
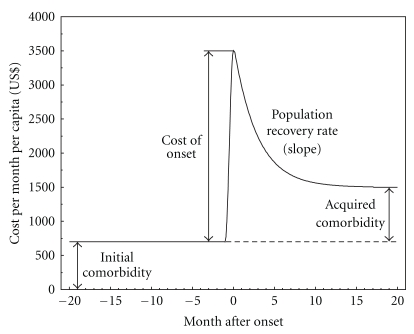
Schematic representation of the pattern of cost per month per capita and the notations for parameters estimated in the four plots below (Figure 3) using dynamic model of changes in medical costs accompanying the onset of chronic disease.

The empirical estimates of the cost trajectories are presented in Figure 1. The shapes of the majority of medical cost trajectories in the time range of 20 months before and after the date of disease onset have the same structure. They can be described in terms of four components sketched in Figure 2. The first one is the pre-diagnosis cost level: this variable measures comorbidity [23] and is referred to as initial comorbidity. The second is the cost of the disease onset. The third variable characterizes the rate of the reduction of medical expenses associated with a disease during the period since diagnosis was made; this variable could be interpreted as a population recovery rate. The fourth variable is the difference between the post and prediagnosis cost levels that characterizes an acquired comorbidity due to a considered disease.

The model for the month patterns of the medical cost trajectories with four respective parameters was constructed as follows. Before the month of disease onset all trajectories demonstrated a plateau; therefore, this region can be described by a single parameter *c* associated with the comorbidity of the studied population group. In the month of onset the trajectories had a sharp peak associated with the cost of onset, which was modeled by a single parameter *P*. During the months after onset, medical costs decreased and the decline was relatively exponential. Therefore, this decline was modeled by an exponential function with a slope *r* characterizing population recovery in terms of medical costs. The level to which the trajectories converge leveling off could also be associated with comorbidity; this level differs from the initial one, *c*, by a quantity *δ* that reflects the contribution of the considered disease to an elevated comorbidity level. Thus, the analytical expression for medical cost per month per capita *C*(m) could be presented as

(1) C(m)=c+(δ+(P−δ)exp (−rm))I(m≥0),

where *m* is the time in months after onset (i.e., time before the onset *m* is negative) and *I*  (*m* ≥ 0) is the indicator function (*I* = 1 for *m* ≥ 0 and *I* = 0 otherwise). The four model parameters correspond exactly to the components presented in Figure 2. Three of them—that is, the pre- and post diagnosis costs associated with initial and acquired comorbidity (*c* and *δ*) and cost of the disease onset *P*—are in U.S. dollars, while the slope of the population recovery rate *r* is in months^−1^. 

**Figure 3:**
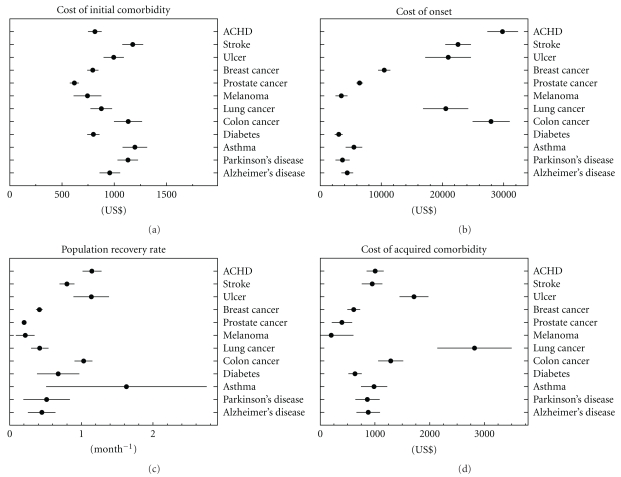
The model parameters (as sketched in Figure 2) were estimated within the 20-month period before and after onset of each of the 12 chronic conditions: (a) cost of initial comorbidity in U.S. dollars, that is, the mean cost per month per capita before onset, (b) cost of onset in U.S. dollars, that is, the mean expenditures in the month of onset, (c) population recovery rate in 1/month, that is, the speed of approaching new steady-state in medical expenditures, and (d) cost of acquired comorbidity in U.S. dollars, that is, an excess in expenditures in a new steady-state compared to those before disease onset. Horizontal bars denote the standard errors of parameter estimates obtained using the nonlinear least squares.

The model was applied to the data and estimated using nonlinear least squares. The resulting curves are presented in Figure 1. The model was estimated for the whole population with the disease onset (see Figure 3) and for subpopulations stratified over other measured variables, for example, disability, comorbidity, or survival after onset. The results of parameter estimates with standard errors for all cases are presented in Tables 2–5 and graphically presented by Supplemental Figures 1(a)–1(d) (see Figures 1(a)–1(d) in supplementary materials available online at doi 10.1155/2011/857892) Comparisons of model estimates allowed us to reveal the properties of the model components described below.

The first component, pre-disease costs level associated with the initial comorbidity, *c*, describes the plateau in the cost trajectories that appeared before the disease onset. In the majority of trajectories this is truly a plateau without a significant time trend. Since only individuals with the disease onset were selected for constructing cost trajectories, the magnitude of the plateau (i.e., the value of the cost per month per capita) reflects the mean comorbidity index measured in terms of medical costs associated with the respective diseases. In other words, the magnitude of the estimates of the initial comorbidity depends on how strongly the risk of the respective disease is determined by comorbidity. The stronger is this association, the higher the mean comorbidity index is in selected individuals. This hypothesis can be tested directly using a separate analysis of subpopulation with the Charlson comorbidity index [19, 20] estimated for a specific month using Medicare information of the previous 12 months. As one can see in Table 2, the positive correlation between the Charlson index and the initial comorbidity is found for all diseases. The strongest associations are detected for stroke, ulcer, lung cancer, and diabetes. Thus, estimates of the initial comorbidities for trajectories generated by different diseases are similar and on average represent mean comorbidity level measured by medical cost. Individual variance of different diseases is moderate and comes from associations of a disease onset with pre-diagnosis comorbidity level. Disability index correlates with comorbidity, so its pattern is similar to comorbidity index. Clear dependence of the first component on the disability index was detected for stroke, diabetes, asthma, ulcer, and ACHD. Dependence on age group was modest; no significant dependence of the initial comorbidity was detected for any disease. For all diseases, except melanoma and colon cancer, the initial comorbidity is larger for those people who died 2.5 years after onset.

The second component, *P*, measures the peak at the date of disease onset (i.e., for month zero in Figure 1): its height reflects disease-specific cost at onset. The order of diseases shown in Figure 1 is based on the decline of this component. High variability of *P* in respect to the specific disease results from the different medical procedures performed at the time of onset (diagnostics and treatment). No significant comorbidity and disability trends were detected for this component. Dependence on age group is also modest, though the difference at the level of 5% significance was detected for ACHD, stroke, lung cancer—for which *P* is larger for those aged 65–80 (i.e., for the younger group)—as well as for diabetes and Parkinson's diseases, for which *P* was lower for younger individuals. For 2.5-year survivors the cost was significantly lower for ACHD, stroke, ulcer, asthma diabetes, and Parkinson's disease, that is, for all diseases except all cancers and Alzheimer's disease. Note that although for some diseases (e.g., asthma, Alzheimer's, ulcer, and melanoma) the increment in cost during several months before onset is visible (likely due to expenses for pre-diagnosis procedures), in the developed version of the model the effect is neglected. In further developments, the cost of onset can be modeled using the normal distribution with finite variance rather than the single parameter *P*.

The third component, *r*, characterizes the rate of reduction of medical expenses associated with a disease during the period since diagnosis was made and is referred to as population recovery rate. This quantity is defined as positive, that is, the larger the estimate of this component the higher the population recovery, or in other words, the faster the decline in medical expenses associated with the disease. Statistically significant estimates of this component were found for all considered diseases (see Figure 3). On the topic of recovery in its clinical meanings, there are certain diseases (e.g., diabetes, Alzheimer's disease) for which the clinical recovery cannot be observed at the individual level. For these diseases the estimate of *r* does not differ significantly from zero. The reduction of medical costs for these diseases (i.e., positive moderate effect of *r*) could be explained by the costs of medical procedures around the time of diagnosis and partial contribution of acute events initiated by the diagnosis onset requiring a specific treatment. The tested associations with comorbidity and disability showed no essential dependences on these indices being detected. Also, no critical dependence on age group was found, though for several diseases (ulcer, colon cancer, diabetes, and Parkinson's disease) the effect was detected at 5% significance level, and for all of these diseases the population recovery for more advanced ages (i.e., 80+) was larger. The population recovery was typically higher for survivors as expected (excluding colon cancer—it had the opposite effect). The high variability in estimates of the component *r* was detected for asthma, diabetes, Parkinson's disease, and Alzheimer's disease, resulting in insignificant associations in comparison of the effects for the two age groups. This set of diseases included primarily the chronic diseases which are defined as the permanent conditions with nonreversible pathologic alterations and generally cannot be completely cured by medications (treatment results in disease remission) [24].

The fourth component, *δ*, represents the acquired comorbidity resulting from the onset of the respective disease (actually, this is the difference between post and prediagnosis cost levels). As one can see in Figure 3, this component is disease specific. A clear positive association of the acquired comorbidity with the disability index was detected for ACHD and prostate cancer. The dependence of acquired comorbidity on the comorbidity index was modest. For some diseases (ACHD and prostate cancer) the correlation was positive, and for several others (e.g., Parkinson's disease) it was the opposite. The latter probably means that the onset of these diseases does not add significant expenditures in case of large initial comorbidity. For the majority of the diseases, a higher age group implied higher acquired comorbidity. For ulcer, this association was significant. However for majority of other diseases it was not (i.e., *P *value is of order  .1−.2). For lung cancer this association was inverse (i.e., lower age group implies higher acquired comorbidity) and significant. As one can expect, for all diseases that the acquired comorbidity was larger for those who died during the first 2.5 years after disease onset. These associations were strongly significant.

## 4. Discussion

In this study, a model was developed capable a quantitative description of the relationships between individual cost trajectories around the onset of an aging-related cancer and noncancer chronic diseases. In total, twelve diseases were analyzed including circulatory diseases (acute coronary heart disease and stroke), cancers (breast, prostate, lung, colon cancers, and melanoma), neurodegenerative diseases (Parkinson's and Alzheimer's diseases), diabetes mellitus, ulcer, and asthma. The main methodological idea was to develop a mathematical model to predict health care costs for these diseases for the time period around the date of the disease onset and create a methodological background for development of forecasting models of dynamic changes of the health state and associated medical costs. The obtained results are important for the whole U.S. elderly population because the diseases included into analysis have high prevalence and high medical costs. Datasets selection was based on the study focus: the trajectories were reconstructed using the NLTCS data linked to the Medicare service use files. This database is nationally representative of the U.S. elderly, so all parameter estimates are supposed to characterize the whole U.S. elderly population (see Supplemental Figure 2 for estimates for 1994 and 1999 cohorts). An innovative approach was developed for selecting the individuals with disease onset and used for identification of the age at onset. We found that the time patterns of the medical costs trajectories were similar for all considered diseases and can be described in terms of four components having the meanings of (i) the pre-diagnosis cost associated with initial comorbidity represented by medical expenditures, (ii) the cost associated with the onset of each disease, (iii) a reduction in medical expenditures after the disease onset, and (iv) the difference between post and prediagnosis cost levels associated with an acquired comorbidity. The description of the trajectories was formalized by a model which explicitly involves four parameters reflecting these four components.

The patterns of medical expenditures evaluated in this paper could help clarify which of the model components is responsible for integrated effects and which of them is more (or less) sensitive to subpopulation specification. Thus, in this paper all medical cost trajectories were considered for the whole population, as well as for the subgroups stratified by disability, comorbidity, age, and survival (for 2.5 years after the onset). The model of medical costs trajectories was applied to all empirically verified patterns, and parameters of the model were statistically estimated and compared. This analysis revealed the basic properties of the medical costs trajectories. The most important of them were the following. The differences in estimates of pre-disease cost level for different diseases were moderate but not identical (Figure 3 and Table 2): since the medical cost trajectories were considered to be conditional on disease-specific incidence, the detected differences reflect variations in disease risk depending on comorbidity. In contrast, the cost of the disease onset was essentially disease specific (Table 3), and the diversity was likely due to the disease-specific diagnostic procedures and initial therapies at the disease onset. The diseases were considered as (i) those with the possible clinical recovery (e.g., ACHD, stroke, and ulcer) and (ii) those with unlikely clinical recovery (e.g., diabetes and Alzheimer's disease). Estimates of population recovery (i.e., the rate of reduction of postdiagnosis cost level) reflect these properties of aging-related diseases. The positive estimates were detected for all diseases; however, the significance of those for diseases with unlikely recovery was lower or absent, especially in subpopulations stratified by disability or comorbidity (Table 4). The acquired comorbidity (i.e., the difference between pre- and postdiagnosis cost levels) was disease specific and strongly depended on the survival status of patients after the onset (Table 5). The parameter estimates (Tables 2–5) confirm that model parameters are chosen so that the effects of multiple diseases on their estimates do not occur or are minimal. The first parameter measures comorbidity before disease onset and represents the effects of multiple comorbidities. The cost of disease onset and acquired comorbidity are defined as the cost level above the mean level of comorbidity. The rate of population recovery, for example, the rate of reduction of medical expenses after a diagnosis, is caused by reduction of costs from the considered disease while changes in the cost due to other diseases are less essential (at least in the first approximation). 

Typically, the medical costs associated with a specific chronic disease were analyzed and projected for a certain period of time after the disease onset or health-related event (e.g., hospitalization) [25]. Often, analyses were performed for specific population groups such as subpopulation of disabled or comorbid individuals [4, 26, 27]. Recently, the ETG approach has been adopted by the Medicare for estimation of disease episode-based medical costs [28]: the detailed information is collected for each disease episode for about 600 clinically homogeneous groups adjusted for patient's severity, age, complications, comorbidities, and major surgeries. Despite being a very useful tool for direct comparison of treatment patterns among providers within the ETG, this approach was not intended to provide the basis for the population-level analysis. Compared to this approach, our method has less details on each disease episode, but allows for inclusion in analysis of all patient-related information on comorbidities (i.e., not only related to one specific disease) and disabilities thus making the whole model more flexible and nondependent on the preselected in the ETG episode-related conditions. In our approach, only data-driven information was incorporated into the model, and the human factor related issues, such as episode-specific information on disease-specific procedures, disabilities, and comorbidities were avoided.

Models of medical costs projections are usually based on the estimated regression models with the majority of independent predictors describing demographic status of the individual, his/her health states, and a level of functional limitations, as well as their interactions [4, 29]. If the health state needs to be described by a number of simultaneously manifested chronic diseases, then the detailed stratification over the respective categorized variables or use of multivariate regression models allows for better description of health states. However, it can result in the abundance of model parameters to be estimated. One way to overcome these difficulties is to use an approach in which the model components would be some demographically based aggregated characteristics allowing to mimic the effects of specific states. 

The model developed in this paper is an example of such an approach: the use of comorbidity index rather than of the set of multiple correlated categorical variables representing the health state allows for essential reduction in the degrees of freedom of the problem. The medical costs of both the first months and the last months of the trajectories investigated in this paper are associated with comorbidity. Since the complete individual trajectory of health changes can be simplified in terms of subsequent incidence events, the developed model of medical costs before and after an incidence event can serve as a building block for constructing the complete individual trajectory. Many uncertainties typical for existing models are overcome with such an approach. Thus, the evaluated model for dynamics of medical costs before and after a chronic disease onset can serve as a key component of a model for projecting the medical expenditures. 

The obtained results are new and important, both substantially and methodologically. Substantially, the evaluated trajectories of medical costs at the disease onset in the U.S elderly provide new information of potential interest for public health expenditures planning and policymakers. This study demonstrated that these trajectories could be described well by the model with four well-defined and interpretable components which were estimated for each of the studied diseases. Interestingly, all studied aging-related cancer and noncancer diseases in elderly had very similar structure of cost trajectories. The model was validated for several population groups and demonstrated a good ability to describe cost trajectories for different levels of disability and comorbidity. There is a useful possibility for this model to be extended to the level of even higher practical importance, such as to forecast health/incidence, mortality, and associated medical costs in the U.S. elderly using even the limited set of parameters (and with a great potential for improvements when more detailed data becomes available), as well as for understanding the currently debated effects of biomedical research, screening, and therapeutic innovations on changes in disease incidence with advancing age.

Methodologically, the developed model brings us to a general microsimulation comprehensive forecasting model of medical expenditures which is formulated as follows. The population dynamics is represented by random trajectories in a covariate space. End of each trajectory is associated with individual death. To simulate an individual trajectory means to evaluate covariates for all time points between beginning age (e.g., 65 years old) and the age of death. During each time point, an individual is under risk of a disease onset and death. The model can be Markov and non-Markov. In the former case, the risks and dynamics are defined by the current health status represented by covariates and age. The model developed in the present paper (or its generalizations) can be used to simulate dynamics of the covariate (i.e., comorbidity index represented by medical cost aggregated during a certain time period) before and after disease onset, and an auxiliary model of the risks of disease onset and mortality associated with the covariate and age has to be attracted to simulate these events. An important property of the model (1) is that it has an input and output represented by the same single quantity: comorbidity measured by medical cost, and this property allows the researchers to use the base model (1) as a building block in simulating the life history as a sequence of such blocks associated with disease onsets. This property also allows for including different chronic diseases into the same approach without increasing the dimensionality of the model. Note that risks of the diseases as well as associations of these risks with potential covariates such as comorbidity, disability indices, and age groups can be roughly estimated using the numbers presented in Table 1 (the detailed investigation of the model for health state projections estimated with Medicare data will be presented in a separate publication).

In many specific cases, averaging over individual trajectories can be performed analytically by reducing the results to aggregated characteristics studied in the present paper and some other quantities observed at the population level. Consider a cohort of individuals under a risk of a certain disease. Let respective survival function *S*(*x*) be known from other studies. This survival function (or corresponding hazard rate *h*(*x*) = −[log *S*(*x*)]_*x*_′ or density function *f*(*x*) = *h*(*x*)*S*(*x*) or probability distribution *F*(*x*) = 1 − *S*(*x*)) can be estimated from Medicare data as well [30, 31]. Assume also that, during the followup, the individuals are not subject to another health event, including death. The medical cost for the cohort of individuals at age *x* can be predicted by summing individual cost trajectories given by (1):

(2) $$\begin{array}{cc} C_{\text{tot}}\left(x\right) & =cS\left(x\right)+\left(c+\delta \right)F\left(x\right)\\ & \;+\left(P-\delta \right)\overset{x}{\underset{0}{\int }}\left(\exp\;\left(-r\left(x-u\right)\right)\right)f\left(u\right)du. \end{array}$$

The first term reflects the contribution of healthy individuals, that is, those that have not developed this disease yet. The mean of their cost is characterized by initial comorbidity *c*, and their fraction equals *S*(*x*). The last two terms characterize the contribution of unhealthy people. They are resulted from integration of individual trajectories *C*(*u*) over different time of onsets denoted by *u*. The second term in (2) describes the acquired comorbidity, and the third term reflects the cost of treatment after onset. The integration can be performed analytically in many specific cases including when (i) the model is characterized by the constant hazard rate, (ii) the cohort of interest is exposed to a specific risk factor (e.g., infection, smoking, or ionizing radiation) with known latent period, and (iii) survival function for a disease is known from empirical analysis, for example, represented by the Kaplan-Meier estimator.

If the general comprehensive microsimulation model is defined as Markov model, the past individual history does not contribute to probabilities of future events or, in other words, current covariates and age have to represent a sufficient set of information for proper description of health state and future event probabilities. By reducing the dimensionality of the model, we are able to better estimate the covariate-specific effect; however, the model becomes less precise. Therefore, the model with a specific set of covariates always represents an approximation to a reality. This is a limitation of used approach, as well as of all Markov models. Specifically, the situation when the model (1) needs to be improved is when the second disease onset occurs almost immediately after the first one. Partly, this can be done using the comprehensive microsimulation model: if the simulation is performed on month-by-month basis, the onset of the second disease can be simulated in any time after onset, including the time period when the recovery is not completed. The higher values of a covariate will provide with higher probabilities of such an event. The approach's precision can be estimated by developing individual trajectories for a pair of disease onsets using the approach close to that described in this paper. Another limitation of the developed modeling approach is that model (1) is not capable of describing all types of diseases equally well: for example, several months before the onset, asthma, Alzheimer's and Parkinson's diseases, and melanoma are not described very well by the model. That could be explained by the diagnostic tests/procedures performed before clinical diagnosis, and therefore these effects were not crucial for the modeling approach. 

At a certain stage of the model development, analytic solutions become no longer possible. Instead, the approach based on microsimulation might be used. Several further generalizations might also be required for improving the comprehensive microsimulation model. One important generalization of the model is an attraction of a model for mortality risks that need to be constructed on assumptions other than those used in model (1). The assumption could include considering a rate of changing cost level as a main predictive variable. Given the model estimated, the simulation of individual trajectories is naturally generalized by considering two competing risks (i.e., the risk of disease onset and the risk of death) which can be dependent or, more specifically, conditionally independent given the value of a covariate (i.e., the medical cost level). Other directions for model generalization could include (i) adjustment to the effect of a second health event that occurred before the complete recovery from the previous one, (ii) adjustment to possible recurrence of the disease diagnosed earlier, (iii) implementation of period and cohort effects, (iv) implementation of generalized models of the risks of the health events with the dependence on the covariate incorporated additional to the dependence on age, (v) incorporation of the effects of increasing medical expenditures before death, and (vi) modeling and implementation of the distribution of the covariate, including the distribution conditional on a specific value in the previous time period. This approach will provide with population projections of health and associated medical costs under the assumption that current tendencies (i.e., those which can be captured by available data) will be held. Specific scenarios regarding the future healthcare environment elaborated by respective panels of experts [7] can also be incorporated into the simulation model. In all these developments models (1) and (2) will serve conveniently in the particular case which must be reproduced numerically or analytically with respective simplifying options of the comprehensive forecasting programs.

## Supplementary Material

Supplementary Figure 1. The model parameters (as sketched in Figure 2), that is, (a) cost of initial comorbidity in US dollars, (b) cost of onset in US dollars, (c) population recovery (slope) in 1/month, and (d) cost of acquired comorbidity in US dollars) estimated in specific groups (in the same sequence as in Tables 2–5): total (black, coincide with results in Figure 3), disability groups (red, the lower point, the higher disability), comorbidity group(blue, the lower point, the higher Charlson index), two age group (violet, <80, 80+), survival status (green, died or nor in 2.5 years after onset).
Supplementary Figure 2. Cohort specific estimates of model parameters (blue for 1994 and red for 1999).

## Figures and Tables

**Table 1:** Characteristics of cohorts followedup for onsets of geriatric diseases.

	ACHD	Stroke	Ulcer	Breast cancer*	Prostate cancer**	Melanoma	Lung cancer	Colon cancer	Diabetes	Asthma	Parkinson's disease	Alzheimer's disease
Total in two waves	34077	34077	34077	20771	13306	34077	34077	34077	34077	34077	34077	34077
Total without prevalent	31785	32807	33485	20241	12597	33970	33945	33752	31615	33503	33836	3386
Onsets	**1375**	**1418**	**384**	**340**	**407**	**89**	**377**	**290**	**791**	**270**	**186**	**374**

Level of IADL/ADL Disability												

Nondisabled	1094	904	265	254	361	68	301	220	616	198	119	209
IADL only or/and 1-2 ADLs	151	211	55	48	31	6	50	45	82	37	27	52
3–6 ADLs	130	303	64	38	15	15	26	25	93	35	40	113

Charlson index												

0	600	576	151	159	231	32	131	127	432	100	86	155
1	298	301	99	85	80	11	105	61	157	80	41	71
2	208	203	49	42	43	18	50	51	102	39	24	57
>2	269	338	85	54	53	28	91	51	100	51	35	91
>2, mean	4.3	4.4	4.4	5.1	5.2	4.2	4.9	5.1	4.2	4.3	4.9	4.1

Age												

≤80	989	815	249	239	315	59	269	183	613	210	115	160
≤80, mean	72	73	73	72	72	73	72	73	71	72	73	75
>80	386	603	135	101	92	30	108	107	178	60	71	214
>80, mean	85	86	85	84	84	84	83	84	86	85	86	86

Survived 2.5 years												

No	340	600	90	62	74	21	289	112	125	57	61	161
Yes	1035	818	294	278	333	68	88	178	666	213	125	213

*Females only.

**Males only.

**Table 2:** Estimates of the initial comorbidity costs in U.S. dollars, *c*.

	ACHD	Stroke	Ulcer	Breast cancer*	Prostate cancer**	Melanoma	Lung cancer	Colon cancer	Diabetes	Asthma	Parkinson's disease	Alzheimer's disease
Total population	**815**	**1176**	**1071**	**795**	**618**	**744**	**876**	**1132**	**800**	**1198**	**1130**	**926**
	±66	±100	±98	±53	±45	±133	±104	±134	±59	±118	±98	±112

Level of IADL/ADL Disability												

Nondisabled	**712**	**824**	**804**	**562**	**592**	**326**	**829**	**974**	**610**	**773**	**853**	**663**
	±58	±79	±75	±54	±44	±53	±103	±123	±55	±102	±114	±97
IADL only or/and 1-2 ADLs	**732**	**1468**	**854**	**636**	**380**	**1550**	**579**	**741**	**650**	**806**	**684**	**782**
	±85	±154	±120	±106	±65	±319	±95	±114	±103	±139	±76	±112
3–6 ADLs	**1604**	**1723**	**1784**	**1079**	**1549**	**735**	**758**	**920**	**1658**	**2733**	**1154**	**1127**
	±143	±108	±225	±129	±151	±193	±134	±223	±153	±291	±159	±144

Charlson index												

0	**414**	**522**	**388**	**329**	**403**	**160**	**327**	**249**	**466**	**478**	**687**	**349**
	±48	±60	±50	±42	±38	±27	±74	±70	±51	±64	±66	±62
1	**909**	**903**	**503**	**941**	**635**	**609**	**714**	**602**	**749**	**868**	**697**	**915**
	±90	±86	±74	±103	±67	±92	±121	±90	±76	±107	±88	±122
2	**719**	**913**	**563**	**292**	**324**	**762**	**712**	**1083**	**750**	**829**	**277**	**886**
	±98	±101	±76	±52	±60	±156	±100	±157	±82	±124	±65	±135
>2	**1447**	**2154**	**2468**	**1296**	**1062**	**627**	**1286**	**716**	**1661**	**2507**	**1988**	**1125**
	±96	±134	±237	±141	±107	±117	±120	±143	±147	±236	±192	±168

Age												

≤80	**770**	**1191**	**1119**	**650**	**598**	**449**	**732**	**1086**	**720**	**1154**	**1095**	**637**
	±64	±107	±113	±55	±46	±86	±106	±163	±50	±103	±120	±123
>80	**927**	**1091**	**901**	**723**	**708**	**687**	**812**	**763**	**855**	**1182**	**980**	**815**
	±76	±94	±82	±112	±82	±94	±111	±121	±92	±196	±123	±84

Survived 2.5 years												

No	**1156**	**1608**	**1772**	**1236**	**923**	**367**	**865**	**382**	**1488**	**1434**	**1139**	**909**
	±109	±145	±234	±157	±123	±84	±111	±67	±178	±206	±146	±125
Yes	**692**	**820**	**692**	**656**	**519**	**757**	**736**	**1237**	**637**	**1037**	**950**	**858**
	±54	±72	±67	±45	±54	±112	±104	±144	±46	±93	±93	±105

*Females only.

**Males only.

**Table 3:** Estimates of the cost of onset in U.S. dollars, *P*.

	ACHD	Stroke	Ulcer	Breast cancer*	Prostate cancer**	Melanoma	Lung cancer	Colon cancer	Diabetes	Asthma	Parkinson's disease	Alzheimer's disease
Total population	**29842**	**22542**	**17849**	**10477**	**6466**	**3482**	**20524**	**27959**	**3045**	**5530**	**3667**	**5323**
	±2513	±2098	±3222	±1017	±544	±977	±3683	±3056	±659	±1362	±1145	±1224

Level of IADL/ADL Disability												

Nondisabled	**30563**	**22778**	**16532**	**8604**	**6710**	**4009**	**21499**	**26792**	**2594**	**4594**	**3960**	**5292**
	±2511	±2301	±2790	±535	±560	±928	±4148	±2365	±641	±1447	±1395	±1348
IADL only or/and 1-2 ADLs	**24485**	**25938**	**21982**	**11108**	**4273**	**6968**	**12573**	**23622**	**3127**	**8383**	**7283**	**6340**
	±3421	±4199	±8714	±2516	±1426	±4072	±2320	±6315	±1239	±2934	±2825	±1578
3–6 ADLs	**28734**	**19711**	**26256**	**14050**	**9916**	**711**	**30138**	**51654**	**7002**	**11164**	**2064**	**4996**
	±3896	±1975	±7450	±3392	±2121	±478	±7767	±28586	±1880	±3861	±1168	±1711

Charlson index												

0	**31622**	**21770**	**15913**	**9489**	**7246**	**3739**	**16684**	**24537**	**3013**	**5469**	**5109**	**4973**
	±3243	±2260	±3838	±806	±722	±1096	±4080	±3276	±753	±1648	±1422	±1147
1	**26803**	**24105**	**8732**	**8685**	**4393**	**3866**	**21846**	**26677**	**2648**	**5688**	**6991**	**3674**
	±2892	±3062	±2556	±846	±696	±1761	±3421	±5773	±647	±1793	±3529	±1136
2	**25813**	**24025**	**25373**	**9813**	**4876**	**3639**	**17694**	**28816**	**2987**	**7046**	**6016**	**3553**
	±5007	±3511	±8658	±1716	±1219	±1504	±3385	±3862	±1816	±2604	±1647	±1154
>2	**29023**	**23317**	**24405**	**7522**	**7526**	**1903**	**18227**	**34630**	**6013**	**5211**	**1897**	**6680**
	±2621	±2289	±5541	±1907	±1265	±646	±3429	±13543	±2207	±2108	±886	±2462

Age												

≤80	**31275**	**24203**	**17059**	**8892**	**6731**	**3734**	**23050**	**24863**	**2615**	**5316**	**1553**	**6517**
	±2598	±2288	±3381	±552	±677	±1220	±4653	±2767	±546	±1253	±367	±2159
>80	**24739**	**19455**	**18669**	**10312**	**5389**	**3547**	**13181**	**34435**	**6193**	**6780**	**7829**	**4462**
	±2627	±2193	±3976	±2859	±983	±876	±2542	±7549	±1797	±2706	±2819	±884

Survived 2.5 years												

No	**37980**	**25559**	**38553**	**10233**	**7061**	**5562**	**20197**	**34916**	**4110**	**11995**	**8860**	**6267**
	±5414	±2967	±9250	±1529	±1498	±3151	±4677	±8162	±1011	±3927	±2950	±1717
Yes	**27690**	**21149**	**13845**	**11284**	**6815**	**2614**	**19755**	**24075**	**2626**	**4098**	**1990**	**4861**
	±1877	±1902	±2484	±1218	±741	±537	±3176	±2379	±536	±1003	±703	±1166

*Females only.

**Males only.

**Table 4:** Estimates of the slope of population recovery rate *r*.

	ACHD	Stroke	Ulcer	Breast cancer*	Prostate cancer**	Melanoma	Lung cancer	Colon cancer	Diabetes	Asthma	Parkinson's disease	Alzheimer's disease
Total population	**1.15**	**0.80**	**1.15**	**0.42**	**0.20**	**0.22**	**0.42**	**1.03**	**0.68**	**1.63**	**0.52**	**1.14**
	±0.13	±0.10	±0.24	±0.05	±0.03	±0.13	±0.12	±0.12	±0.29	±1.12	±0.32	±0.66

Level of IADL/ADL Disability												

Nondisabled	**1.14**	**0.78**	**1.42**	**0.37**	**0.21**	**0.32**	**0.45**	**1.06**	**0.82**	**1.79**	**0.45**	**0.98**
	±0.12	±0.11	±0.29	±0.04	±0.03	±0.12	±0.13	±0.13	±0.51	±1.82	±0.30	±0.64
IADL only or/and 1-2 ADLs	**0.95**	**1.01**	**2.59**	**0.37**	**0.67**	**0.51**	**0.16**	**0.92**	**0.21**	**0.59**	**1.78**	**0.19**
	±0.21	±0.17	±0.65	±0.16	±0.57	±0.60	±0.07	±0.26	±0.15	±0.27	±1.59	±0.11
3–6 ADLs	**1.36**	**0.68**	**0.85**	**0.56**	**2.06**	**0.11**	**0.64**	**2.10**	**0.67**	**1.10**	**0.09**	**14.19**
	±0.25	±0.10	±0.22	±0.14	±1.22	±0.34	±0.20	±0.75	±0.31	±0.61	±0.25	>100

Charlson index												

0	**1.21**	**0.78**	**1.36**	**0.38**	**0.22**	**0.23**	**0.31**	**0.91**	**0.86**	**1.05**	**1.66**	**0.29**
	±0.15	±0.09	±0.37	±0.06	±0.04	±0.11	±0.14	±0.17	±0.40	±0.48	±0.99	±0.19
1	**1.04**	**0.85**	**0.29**	**0.33**	**0.17**	**0.82**	**0.49**	**0.63**	**0.37**	**2.04**	**3.20**	**1.07**
	±0.14	±0.14	±0.10	±0.05	±0.06	±0.71	±0.11	±0.12	±0.19	±1.80	±3.05	±0.91
2	**0.77**	**1.27**	**1.92**	**0.52**	**0.21**	**0.23**	**0.38**	**0.85**	**0.68**	**1.65**	**0.16**	**0.07**
	±0.19	±0.27	±0.68	±0.14	±0.13	±0.16	±0.14	±0.13	±0.61	±0.87	±0.10	±0.15
>2	**1.26**	**0.74**	**1.43**	**0.32**	**0.30**	**0.15**	**0.31**	**1.63**	**1.17**	**0.92**	**0.13**	**18.87**
	±0.17	±0.10	±0.45	±0.09	±0.12	±0.45	±0.10	±0.60	±0.91	±0.63	±0.19	>100

Age												

≤80	**1.13**	**0.84**	**0.94**	**0.34**	**0.20**	**0.20**	**0.44**	**0.88**	**0.50**	**1.46**	**0.01**	**1.49**
	±0.12	±0.11	±0.21	±0.04	±0.04	±0.16	±0.14	±0.12	±0.23	±0.88	±0.00	±1.21
>80	**1.23**	**0.71**	**1.54**	**0.44**	**0.22**	**0.27**	**0.34**	**1.44**	**2.81**	**2.90**	**0.88**	**0.71**
	±0.16	±0.11	±0.33	±0.14	±0.08	±0.12	±0.09	±0.30	±1.71	±6.48	±0.58	±0.41

Survived 2.5 years												

No	**1.04**	**0.75**	**1.13**	**0.34**	**0.20**	**0.33**	**0.48**	**1.65**	−0.01	**2.08**	**0.56**	**1.55**
	±0.24	±0.15	±0.37	±0.15	±0.17	±0.75	±0.23	±0.45	±0.01	±2.42	±0.32	±2.21
Yes	**1.28**	**0.92**	**2.05**	**0.50**	**0.28**	**0.21**	**0.58**	**0.85**	**0.66**	**1.51**	**2.60**	**1.53**
	±0.12	±0.10	±0.40	±0.05	±0.04	±0.08	±0.12	±0.09	±0.22	±1.01	±5.34	±0.77

*Females only.

**Males only.

**Table 5:** Estimates of the Acquired Comorbidity Costs in U.S. dollars, *δ*.

	ACHD	Stroke	Ulcer	Breast cancer*	Prostate cancer**	Melanoma	Lung cancer	Colon cancer	Diabetes	Asthma	Parkinson's disease	Alzheimer's disease
Total population	**1005**	**949**	**630**	**613**	**399**	**201**	**2818**	**1288**	**637**	**985**	**864**	**1244**
	±157	±189	±204	±116	±185	±410	±681	±230	±124	±242	±223	±251

Level of IADL/ADL Disability												

Nondisabled	**846**	**977**	**657**	**662**	**293**	**309**	**2913**	**1507**	**729**	**959**	**777**	**1109**
	±134	±173	±167	±137	±168	±174	±685	±226	±138	±213	±260	±256
IADL only or/and 1-2 ADLs	**1779**	**693**	**362**	**1095**	**1360**	**2036**	−331	**690**	−11	**419**	**1749**	**347**
	±339	±294	±211	±416	±326	±772	±1085	±235	±433	±321	±247	±663
3–6 ADLs	**1673**	**1347**	**467**	**423**	**2917**	−371	**1053**	**2289**	**470**	**1016**	−521	**1477**
	±325	±245	±479	±240	±471	±1306	±416	±625	±273	±597	±3671	±344

Charlson index												

0	**702**	**868**	**696**	**908**	**347**	**117**	**2779**	**1321**	**535**	**838**	**1140**	**1024**
	±119	±150	±175	±188	±185	±240	±935	±238	±111	±196	±172	±365
1	**898**	**1061**	**329**	**173**	**272**	**678**	**1401**	**923**	**447**	**1394**	**1076**	**874**
	±179	±207	±209	±197	±354	±283	±478	±227	±180	±257	±215	±240
2	**974**	**2332**	**1180**	**672**	**638**	−144	**2474**	**771**	**417**	**215**	**106**	−28
	±263	±308	±303	±188	±494	±435	±951	±293	±194	±261	±938	±4125
>2	**1357**	**657**	−431	**253**	**1568**	**886**	**2211**	**2423**	**1107**	**201**	−423	**1198**
	±239	±239	±348	±268	±444	±1024	±734	±521	±345	±363	±1174	±370

Age												

≤80	**934**	**860**	**338**	**705**	**242**	**518**	**3436**	**1122**	**644**	**997**	**NA^#^**	**1613**
	±149	±186	±210	±141	±219	±517	±811	±246	±117	±220		±336
>80	**1227**	**1068**	**1002**	**364**	**581**	**42**	**506**	**1432**	**895**	**555**	**1343**	**1183**
	±198	±223	±210	±238	±323	±226	±335	±296	±198	±352	±320	±233

Survived 2.5 years												

No	**3836**	**2444**	**2856**	**2768**	**2117**	**2857**	**5288**	**3687**	**NA^#^**	**2895**	**1591**	**3067**
	±616	±425	±900	±563	±1086	±1144	±1066	±584		±725	±485	±518
Yes	**767**	**785**	**646**	**533**	**392**	−140	**721**	**410**	**448**	**730**	**574**	**667**
	±106	±127	±143	± 99	±141	±240	±267	±185	± 86	±174	±168	±179

*Females only.

**Males only.

^#^Parameter is not identified because of small respective estimate of *r*. The model *C*(m) = *c* + *P* · *I*  (*m* ≥ 0) needs to be used instead of that given in (1).
