# Supplementary material for: Medical Cost Trajectories and Onsets of Cancer and NonCancer Diseases in US Elderly Population
Source: Comput Math Methods Med. 2011 Jun 1;2011:857892. doi: 10.1155/2011/857892 (PMC3115464; doi:10.1155/2011/857892)
Supplement: Supplementary file 1 — Supplementary Figure 1. The model parameters (as sketched in Figure 2), that is, (a) cost of initial comorbidity in US dollars, (b) cost of onset in US dollars, (c) population recovery (slope) in 1/month, and (d) cost of acquired comorbidity in US dollars) estimated in specific groups (in the same sequence as in Tables 2–5): total (black, coincide with results in Figure 3), disability groups (red, the lower point, the higher disability), comorbidity group(blue, the lower point, the higher Charlson index), two age group (violet, <80, 80+), survival status (green, died or nor in 2.5 years after onset). Supplementary Figure 2. Cohort specific estimates of model parameters (blue for 1994 and red for 1999). [file 857892.f1.pdf]

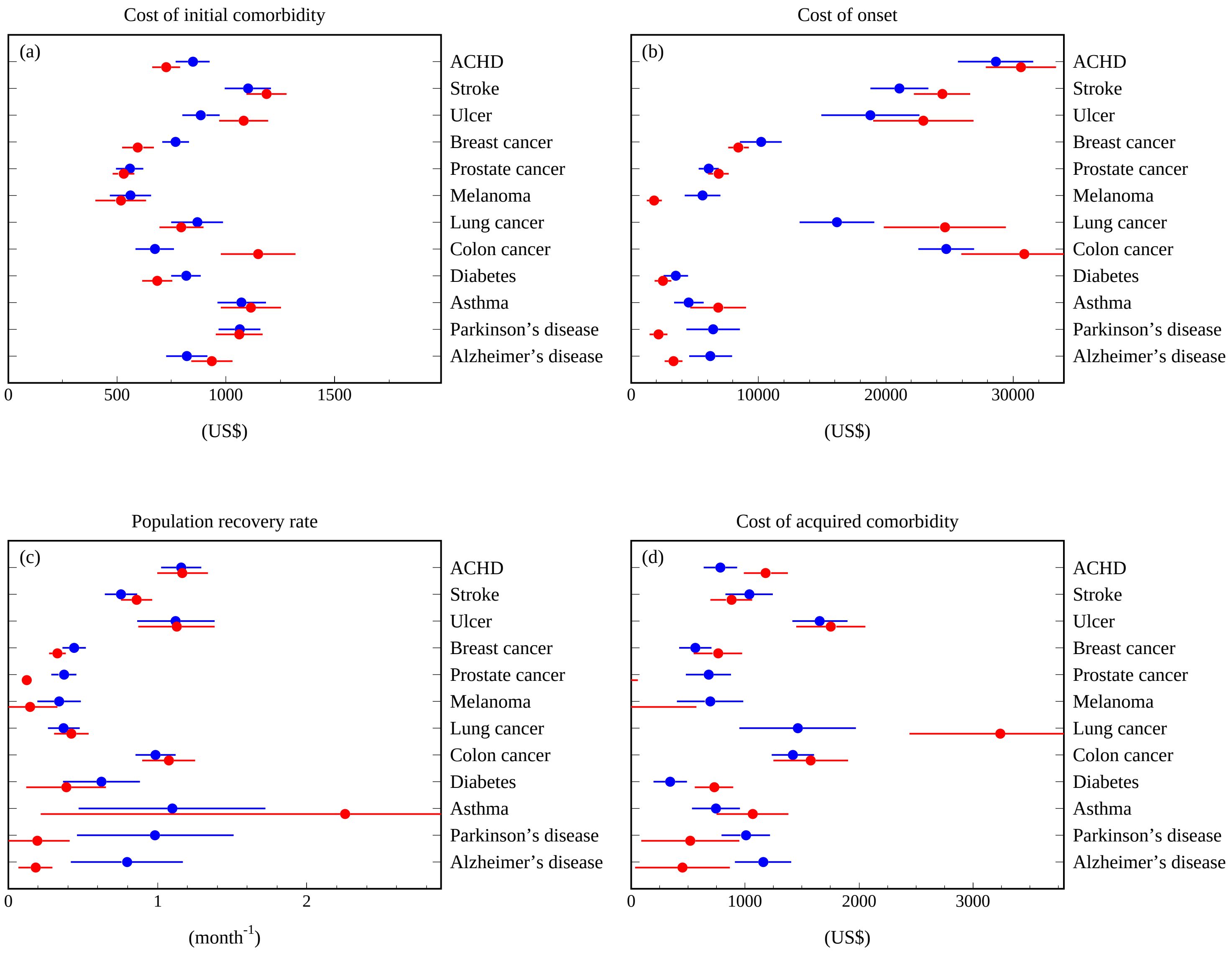

Cost of initial comorbidity
(a)
ACHD
Stroke
Ulcer
Breast cancer
Prostate cancer
Melanoma
Lung cancer
Colon cancer
Diabetes
Asthma
Parkinson’s disease
Alzheimer’s disease
0
500
1000
1500
(US$)
Cost of onset
(b)
0
10000
20000
30000
(US$)
Population recovery rate
(c)
0
1
2
(month$^{-1}$)
Cost of acquired comorbidity
(d)
0
1000
2000
3000
(US$)
