# Supplementary material for: Medical Cost Trajectories and Onsets of Cancer and NonCancer Diseases in US Elderly Population
Source: Comput Math Methods Med. 2011 Jun 1;2011:857892. doi: 10.1155/2011/857892 (PMC3115464; doi:10.1155/2011/857892)
Supplement: Supplementary file 2 [file 857892.f2.pdf]

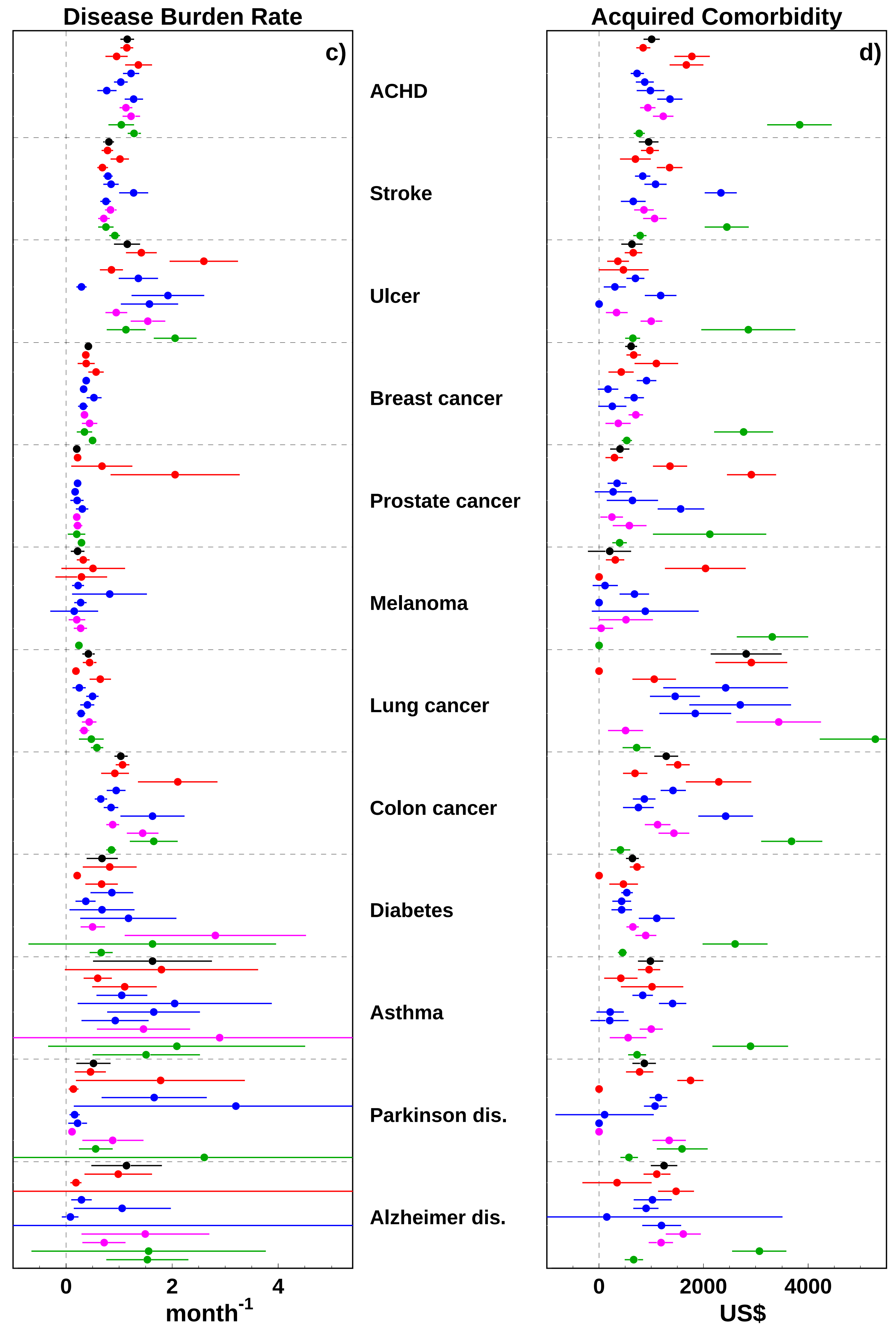

Disease Burden Rate
Acquired Comorbidity
c)
d)
ACHD
Stroke
Ulcer
Breast cancer
Prostate cancer
Melanoma
Lung cancer
Colon cancer
Diabetes
Asthma
Parkinson dis.
Alzheimer dis.
0
2
4
month^{-1}
0
2000
4000
US$
