# Supplementary material for: Medical Cost Trajectories and Onsets of Cancer and NonCancer Diseases in US Elderly Population
Source: Comput Math Methods Med. 2011 Jun 1;2011:857892. doi: 10.1155/2011/857892 (PMC3115464; doi:10.1155/2011/857892)
Supplement: Supplementary file 3 [file 857892.f3.pdf]

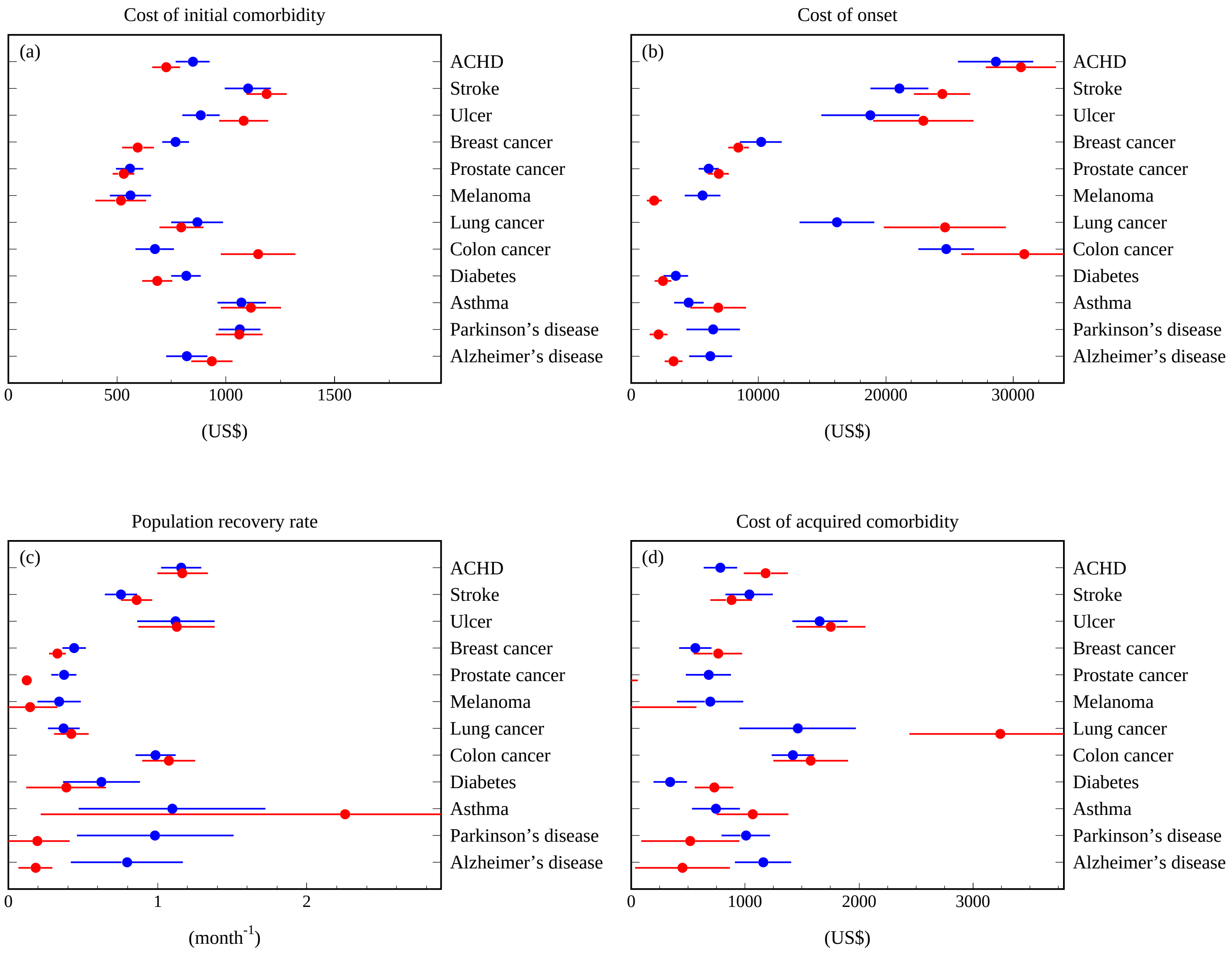

Cost of initial comorbidity
(a)
ACHD
Stroke
Ulcer
Breast cancer
Prostate cancer
Melanoma
Lung cancer
Colon cancer
Diabetes
Asthma
Parkinson's disease
Alzheimer's disease
0
500
1000
1500
(US$)
Cost of onset
(b)
0
10000
20000
30000
(US$)
Population recovery rate
(c)
0
1
2
(month-1)
Cost of acquired comorbidity
(d)
0
1000
2000
3000
(US$)
